# Inhibitory and Injury-Protection Effects of O-Glycan on Gastric Epithelial Cells Infected with Helicobacter pylori

**DOI:** 10.1128/iai.00393-22

**Published:** 2022-10-03

**Authors:** Yuzuo Chen, Zhihui Tang, Lifa Fu, Renjie Liu, Lu Yang, Baoning Wang

**Affiliations:** a Department of Microbiology, West China School of Basic Medical Sciences and Forensic Medicine, Sichuan University, Chengdu, China; University of California San Diego School of Medicine

**Keywords:** *Helicobacter pylori*, O-Glycan, cholesterol-α-glucosyltransferase, gastric cancer

## Abstract

Helicobacter pylori (H. pylori) is an important pathogen that can cause gastric cancer. Multiple adhesion molecules mediated H. pylori adherence to cells is the initial step in the infection of host cells. H. pylori cholesterol-α-glucosyltransferase (CGT) recognizes and extracts cholesterol from cell membranes to destroy lipid raft structure, further promotes H. pylori adhesion to gastric epithelial cells. O-Glycan, a substance secreted by the deep gastric mucosa, can competitively inhibit CGT activity and may serve as an important factor to prevent H. pylori colonization in the deep gastric mucosa. However, the inhibitory and injury-protection effects of O-Glycan against H. pylori infection has not been well investigated. In this study, we found that O-Glycan significantly inhibited the relative urease content in the coinfection system. In the presence of O-glycan, the injury of GES-1 cells in H. pylori persistent infection model was attenuated and the cell viability was increased. We use fluorescein isothiocyanate-conjugated cholera toxin subunit B (FITC-CTX-B) to detect lipid rafts on gastric epithelial cells and observed that O-glycan can protect H. pylori from damaging lipid raft structures on cell membranes. In addition, transcriptome data showed that O-glycan treatment significantly reduced the activation of inflammatory cancer transformation pathway caused by H. pylori infection. Our results suggest that O-Glycan is able to inhibit H. pylori persistent infection of gastric epithelial cells, reduce the damage caused by H. pylori, and could serve as a potential medicine to treat patients infected with H. pylori.

## INTRODUCTION

**FIG 1:**
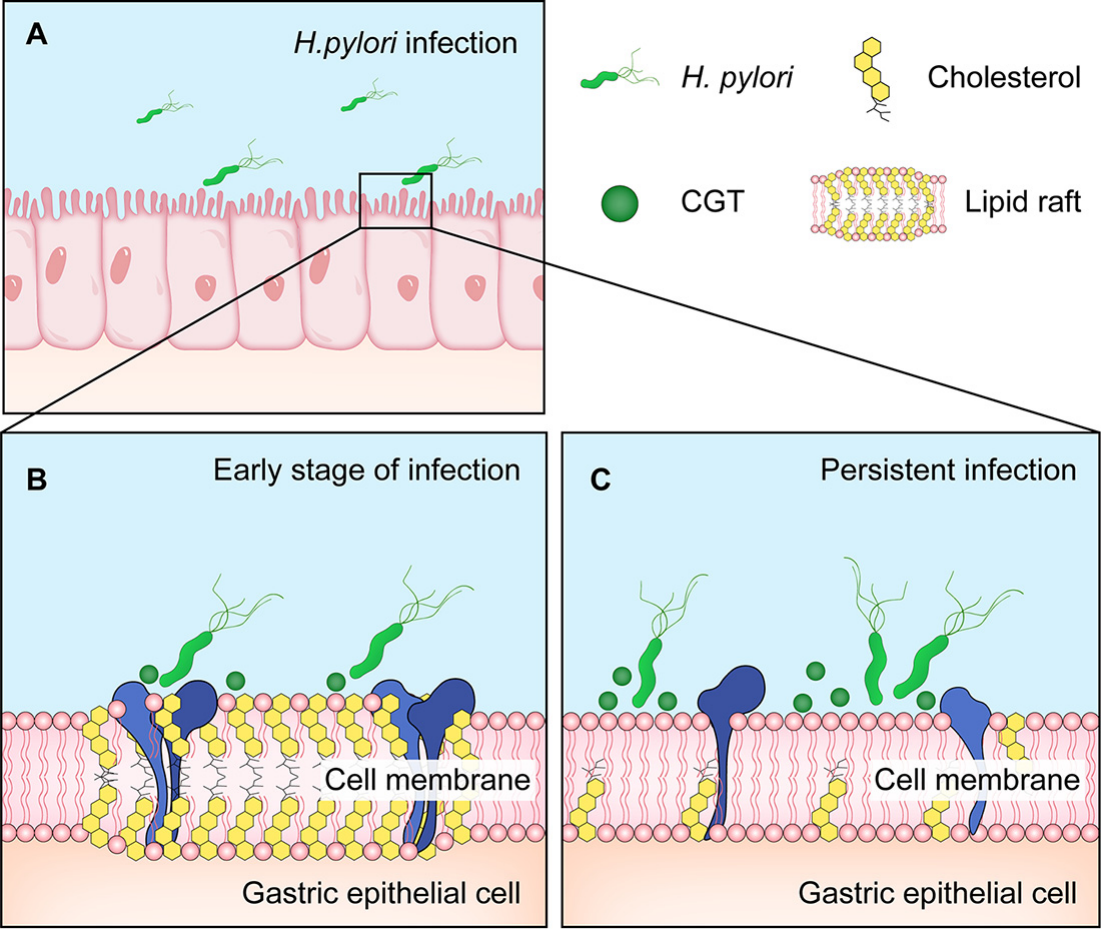
Effects of on host–pathogen interaction. (A) H. pylori infects gastric epithelial cells. (B) In the early stage of infection, CGT promotes the recruitment of binding antigens in lipid rafts and further enhances the colonization of H. pylori. (C) In the persistent infection stage, H. pylori infection disrupts lipid rafts of gastric epithelial cells.

Helicobacter pylori infection is associated with a high incidence of gastrointestinal diseases, including gastritis, peptic ulcers, and gastric cancer (1). The population infected with H. pylori suffers a 2- to 8-fold higher risk of gastric cancer compared to those free of infection (2). As the first and important step of the H. pylori–related pathogenicity, a variety of adhesion molecules mediate H. pylori colonization on the surface of gastric epithelial cells (3). For example, Le^b^ blood group antigen binding adhesin (BabA) (4), sialic acid-binding adhesin (SabA) (5), and outer membrane adhesin (HopQ) (6) bind to adhesion factors related to Lewis antigens, gangliosides, and carcinoembryonic antigens on the surface of human gastric epithelial cells, respectively (7–9). In the early stage of infection, H. pylori was reported to secret cholesterol-α-glucosyltransferase (CGT) interacts with specialized cholesterol-rich membrane microdomains, known as lipid rafts (10), thereby promoting the recruitment of binding antigens in lipid rafts and further enhancing the colonization of H. pylori (Fig. 1A and B) (11, 12).

CGT extracts cholesterol from host cell membrane to generate cholesterol glycosides (13). H. pylori persistent infection result in depletion of cholesterol from host membranes, disrupting lipid rafts, which is closely associated with immune escape (Fig. 1C) (10, 14). Therefore, GGT is considered a promising target for drugs aiming at the eradication of H. pylori infection. Kawakubo et al. found that O-Glycan capped by α1,4-linked *N-acetylglucosamine* (α1,4-GlcNAc), a substance secreted by the deep gastric mucosa, can competitively inhibit CGT activity and may serve as an important factor to prevent H. pylori colonization in the deep gastric mucosa (15). CGT enhances H. pylori adhesion, meanwhile, persistent infection leads to the disruption of lipid rafts. O-Glycan can competitively inhibit the activity of CGT, however, the mechanism of action for O-Glycan against H. pylori infection requires further exploration.

This study observed that O-Glycan inhibited H. pylori adhesion to gastric epithelial cells, which is helpful to attenuate H. pylori-induced injury to the host. In addition, O-Glycan inhibited H. pylori-induced disruption of the lipid rafts of the gastric epithelial cell membrane and suppress the inflammation-cancer transformation, which attenuated H. pylori-induced injury to the gastric epithelium. This study clarifies that O-Glycan may play a role as an antibacterial substance in inhibiting H. pylori-induced injury to gastric epithelial cells and the progression of gastric cancer.

## RESULTS

### O-Glycan reduction of relative urease content of H. pylori in persistently infected GES-1 gastric epithelial cells.

**FIG 2:**
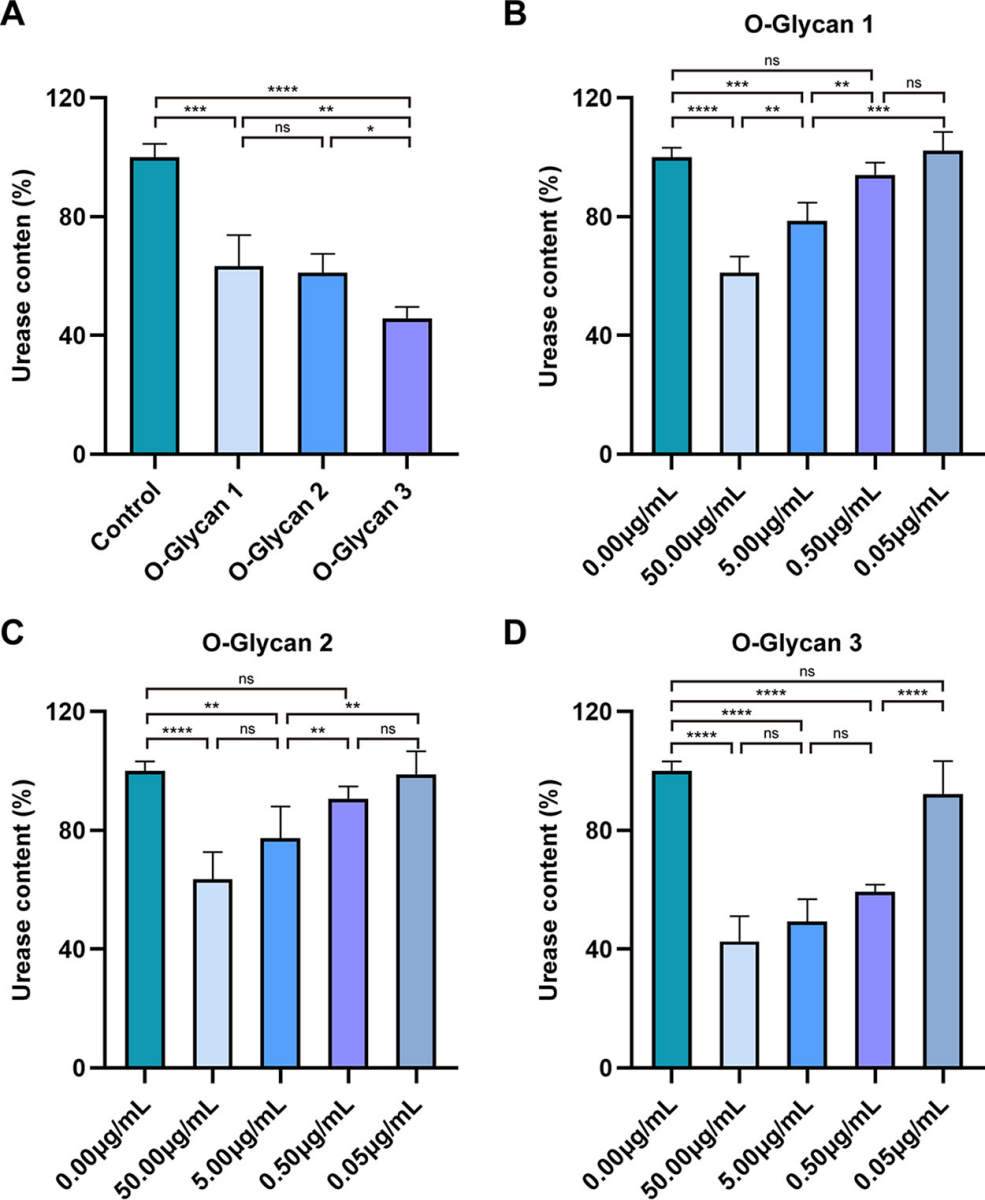
O-Glycan reduction of relative urease content (%) of H. pylori in persistently infected GES-1 cells. (A) In the *in vitro* infection model, all 3 types of O-glycans significantly inhibited the relative urease content of H. pylori (ATCC 43504) at 50.00 μg/mL, and especially, the relative urease content was most significantly reduced to 42.66% after the treatment with O-Glycan 3. (B) The relative urease content of H. pylori (ATCC 43504) decreased to 61.19%, 78.61%, 94.12% and 102.27% after treatment with O-Glycan 1 at 50.00 μg/mL, 5.00 μg/mL, 0.50 μg/mL, and 0.05 μg/mL, respectively. (C) The relative urease content of H. pylori (ATCC 43504) decreased to 63.52%, 77.35%, 90.68% and 98.97% after treatment with O-Glycan 2 at 50.00 μg/mL, 5.00 μg/mL, 0.50 μg/mL, and 0.05 μg/mL, respectively. (D) The relative urease content of H. pylori (ATCC 43504) decreased to 42.66%, 49.39%, 59.36% and 92.34% after treatment with O-Glycan 3 at 50.00 μg/mL, 5.00 μg/mL, 0.50 μg/mL, and 0.05 μg/mL, respectively. The experiments were repeated four times for all groups, statistical significance was calculated using a one-way ANOVA test, followed by the Tukey’s methods. *, *P < *0.05; **, *P < *0.01; ***, *P < *0.001; ****, *P < *0.0001; ns, not significant.

According to Kawakubo et al., O-Glycan contained in mucins secreted by gastric gland cells exerted the effects to inhibit H. pylori and limit its colonization in the deep gastric gland cells (15). The urease produced by H. pylori generates ammonia by decomposing urea, which allows H. pylori to maintain the surrounding pH value close to neutral in the gastric acid environment, thereby facilitating the survival of H. pylori in the stomach and its colonization in the gastric mucosa (16). Clinically, rapid urease test is commonly used to detect whether patients are infected with H. pylori (17). In order to further determine the effect of O-Glycan on H. pylori infection, we treated GES-1 gastric epithelial cells with 3 types of O-glycans, followed by infection with H. pylori strains ATCC 43504 for 24 h, respectively, to determine their effects on the relative urease content of H. pylori. As shown in Fig. 2A, Urease levels were significantly reduced in all 3 types of O-Glycan treatment groups. Especially, there was a statistical significance in the inhibitory effect of O-Glycan 3 (*P < *0.05). Surprisingly, similar effects were observed in GES-1 cells infected with SS1 strains (Fig. S1).

In order to establish the optimal concentration of O-Glycan, the degree of inhibition on the urease content of H. pylori (ATCC 43504) with 3 types of O-glycans at various concentrations was determined as per the procedures in the previous experiment. The relative urease content of H. pylori decreased significantly with the increased concentration of O-Glycan. The results showed that O-Glycan 1 and O-Glycan 2 reduced urease levels significantly only at 50.00 μg/mL and 5.00 μg/mL (Fig. 2B and C). However, O-Glycan 3 could significantly reduce urease content at concentrations of 50.00 μg/mL, 5.00 μg/mL and 0.50 μg/mL (Fig. 2D). To optimize the dose of O-Glycan, we chose O-Glycan 3 with a concentration of 0.50 μg/mL for subsequent experiments. This study revealed the inhibitory effect of O-Glycan on the urease content of H. pylori with an *in vitro* infection model, suggesting that O-Glycan is potential for the treatment of H. pylori infection (18).

### O-Glycan inhibition of injuries in GES-1 cells with persistent infection with H. pylori.

**FIG 3:**
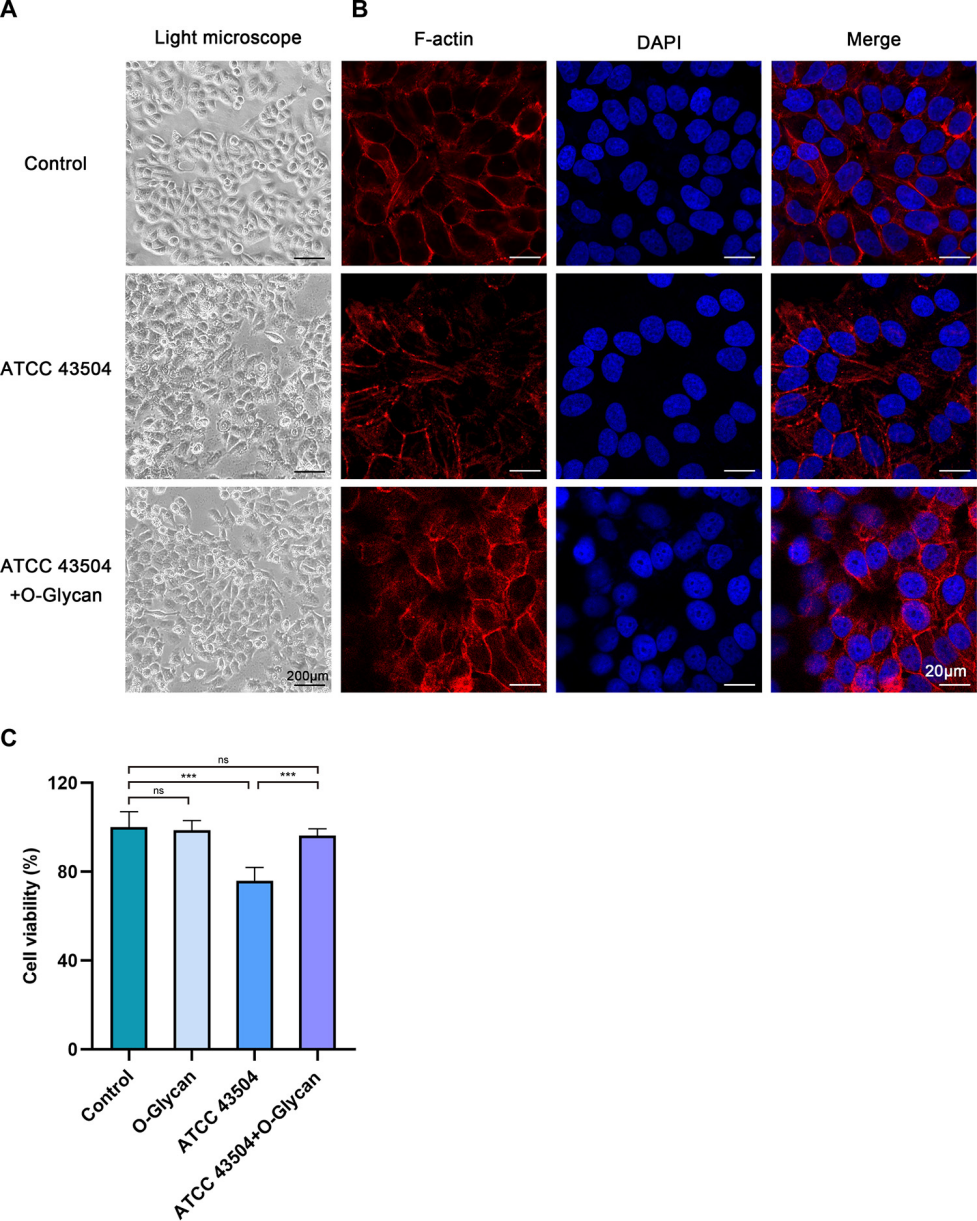
O-Glycan inhibited the injuries of GES-1 cells due to persistent H. pylori infection. (A) Morphological observations at 24 h under the light microscope for normal GES-1 cells, ATCC 43504-infected GES-1 cells, and ATCC 43504-infected GES-1 cells treated with O-Glycan. Scale bar: 200 μm. (B) The cytoskeleton was labeled with F-actin (Red) and the nuclear chromatin was labeled with DAPI (Blue). Fluorescence staining of GES-1 cells in each group was analyzed under the confocal microscope. Scale bar: 20 μm. (C) Cell viability of GES-1 after with infected ATCC 43504 and treatment of O-Glycan or not was detected by CCK-8 assay. ***, *P < *0.001; ns, not significant.

Persistent H. pylori stimulation of gastric epithelial cells leads to changes in cell morphology and cell viability, which further affects gastric epithelial cell injury (19). To explore the effect of persistent H. pylori infection on the morphology of GES-1 cells and the protective effect of O-Glycan on the cell morphology, we observed the morphology of H. pylori (ATCC 43504)-infected GES-1 cells at 24 h under the light microscope. As demonstrated in Fig. 3A, elongation of GES-1 cells was observed after H. pylori infection, cell membrane boundary is not clear, characterized by a serious morphological damage (20). In contrast, the morphology of GES-1 cells in the O-Glycan treatment group was relatively normal. To further visualize the morphology of GES-1 cells, immunofluorescence staining was employed to evaluate the morphology of GES-1 cells under the confocal microscope. As exhibited in Fig. 3B, GES-1 cells infected with H. pylori experienced morphological changes, characterized by deformed nuclei and elongated cytoskeleton. For GES-1 cells treated with the O-Glycan, the intact nucleus and clear cytoskeleton were observed. Similar results were obtained with SS1-infected GES-1 cells (Fig. S2A to B). After light microscope images were collected, CCK-8 assay were determined to quantitatively assess cell viability. No significant reduction in cell viability was observed in the presence of O-Glycan alone or in the presence of both H. pylori (ATCC 43504) and O-Glycan, but a large reduction in cell viability was observed only in the presence of ATCC 43504 (Fig. 3C). Moreover, a small reduction in cell viability was observed in the presence of SS1 (Fig. S2C). These data indicated that O-Glycan improved the injury and morphological changes of gastric epithelial cells induced by persistent H. pylori infection.

### O-Glycan inhibition of H. pylori adhesion to GES-1 cells.

**FIG 4:**
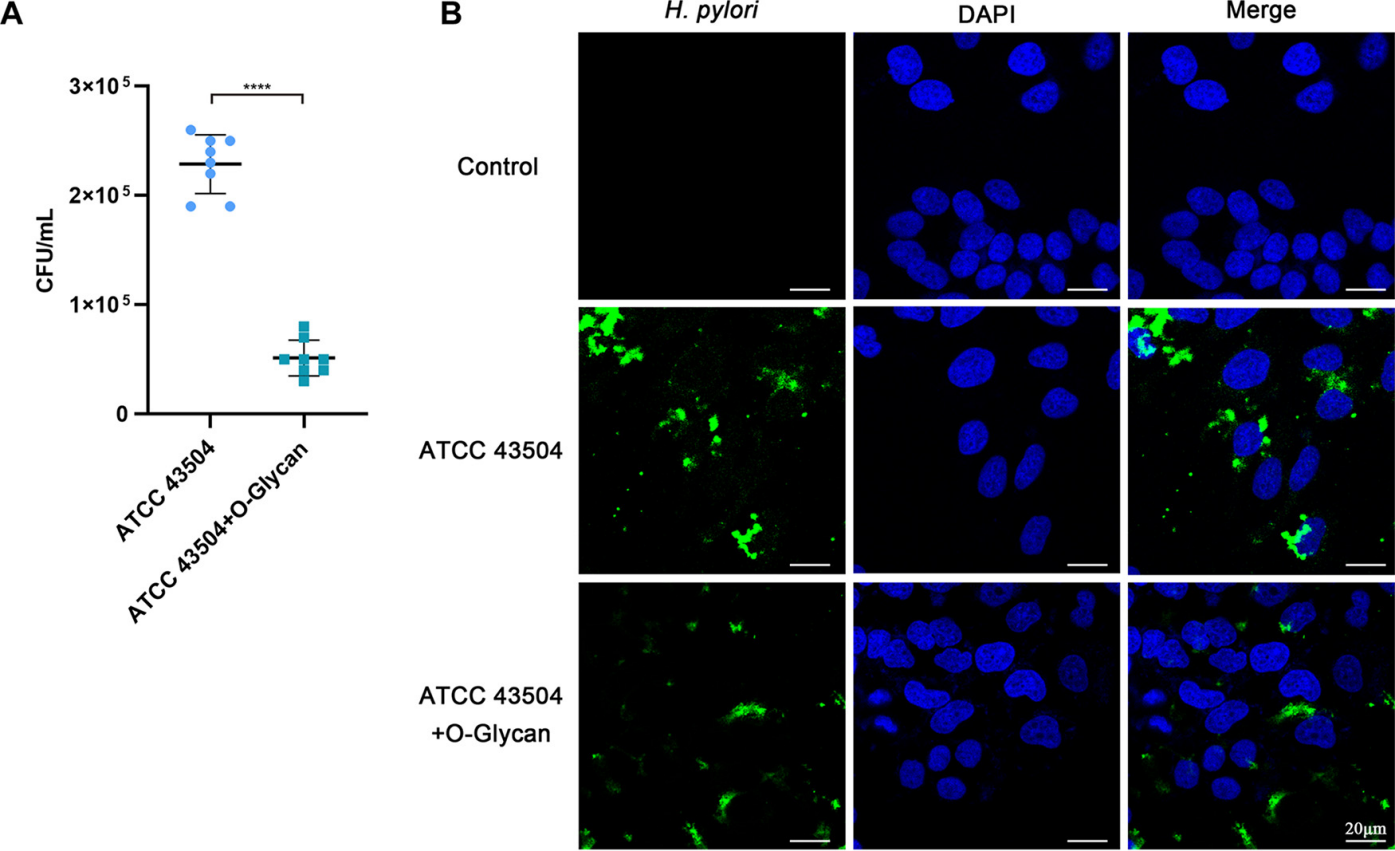
O-Glycan inhibition of H. pylori adhesion to GES-1 cells. GES-1 cells were untreated or treated with 0.50 μg/mL O-Glycan and then infected with TACC 43504 at an MOI of 100 for 24 h. (A) Viable H. pylori count was performed to evaluate its adhesion to GES-1 cells. Data correspond to three independent experiments; The Student's *t* test was used to analyze the statistically significant difference between groups. ****, *P < *0.0001. (B) ATCC 43504 were labeled with anti-H. pylori antibody (Green) and nuclei were labeled with DAPI (Blue). Fluorescence signals were analyzed using the confocal microscope. Scale bar: 20 μm.

H. pylori adhesion to cells is the first step in host cell infection. Previous studies have revealed that H. pylori adheres to and colonizes host cells through CGT, leading to the development of disease (10, 11). In order to investigate the effect of O-Glycan on H. pylori adhesion to gastric epithelial cells, gastric epithelial cells were treated with O-Glycan before infection, and the activity of H. pylori adhesion to gastric epithelial cells was assessed by viable H. pylori count 24 h after infection (Fig. S3A). The activity of H. pylori adhesion to gastric epithelial cells was significantly compromised after the treatment with O-Glycan, as seen in Fig. 4A. The confocal microscope was then used to spot the location of H. pylori in infected cells and the result was equivalent to that of viable H. pylori count. Fig. 4B reveals that the number of H. pylori adhered to GES-1 cells treated with O-Glycan was far fewer than those untreated with O-Glycan. Similarly, we also observed similar results using the SS1 strains (Fig. S3). These results indicated that O-glycan could inhibit the adhesion of H. pylori to gastric epithelial cells.

### Protective effect of O-Glycan on lipid rafts of GES-1 cells infected with H. pylori.

**FIG 5:**
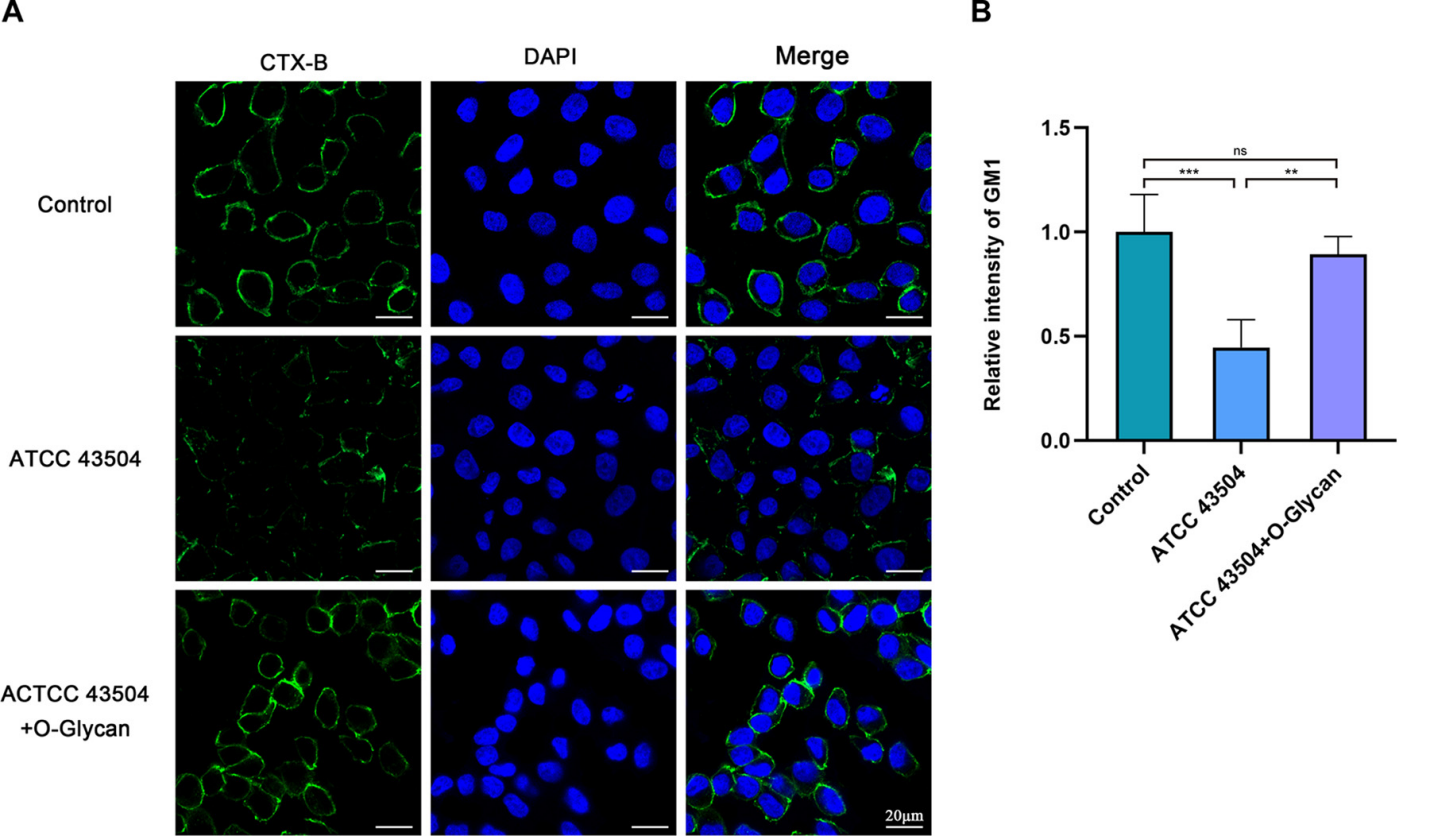
Protective effect of O-Glycan on lipid rafts of GES-1 cells infected with H. pylori. GES-1 cells were untreated or treated with 0.50 μg/mL O-Glycan and then infected with ATCC 43504 at an MOI of 100 for 24 h. (A) Confocal microscopic analysis of the lipid raft marker GM1 (stained with CTX-B, green) in cellular membranes of cells and DAPI to visualize cell nuclei (blue). Scale bar: 20 μm. (B) Quantitative measurements of relative superficial GM1 signal. For image analyses, was selected from 4 experiments and analyzed by ImageJ software. **, *P < *0.01; ***, *P < *0.001; ns, not significant.

H. pylori adheres to gastric epithelial cells via CGT and compromises lipid rafts on the cell membrane, thereby facilitating the immune escape of H. pylori and causing persistent infection (10, 11, 14). Further, we explored the inhibitory effect of O-Glycan on H. pylori-induced disruption of lipid rafts on the gastric epithelial cell membrane. According to the results of immunofluorescence staining (Fig. 5 and Fig. S4), without the treatment with O-Glycan, the ganglioside GM1 (a constitutive marker of lipid rafts) on the membrane surface of the H. pylori-infected GES-1 cells was largely lost, while that on GES-1 cells treated with O-Glycan was relatively intact. These results unveiled that O-Glycan protected the lipid rafts on the membrane surface of H. pylori-infected cells and might play an important role in suppressing the immune escape of H. pylori.

### O-Glycan inhibition of upregulation of H. pylori infection-mediated inflammation-cancer transformation pathway in GES-1 cells.

**FIG 6:**
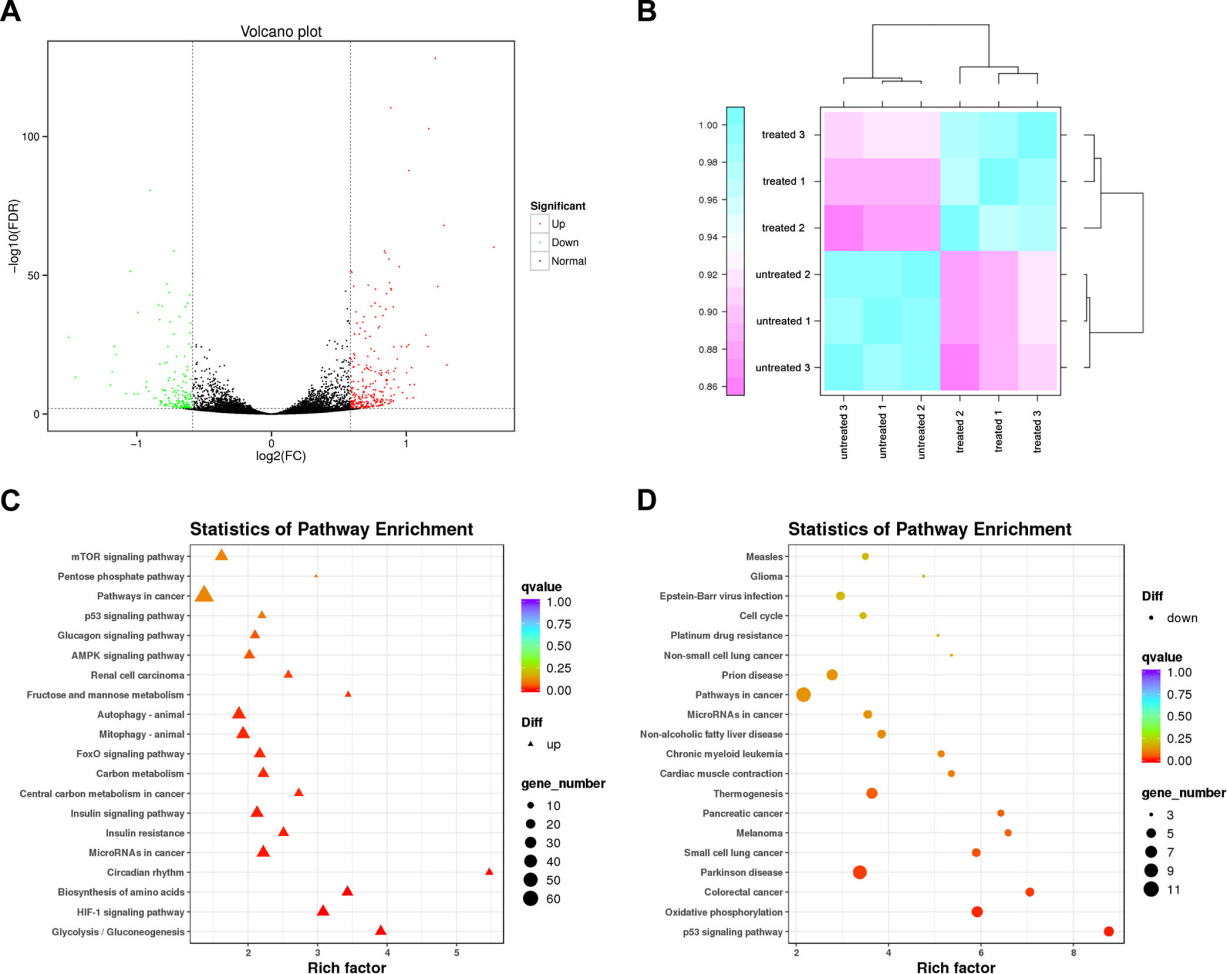
O-Glycan inhibition of upregulation of H. pylori infection-mediated inflammation-cancer transformation pathway in GES-1 cells. (A) Differential gene expression profiles in *H pylori*-infected GES-1 cells treated or untreated with O-Glycan, with upregulated genes marked in red and downregulated genes marked in green. (B) Heatmap of sample expression correlation. Pearson's Correlation Coefficient R was used as an evaluation index of biological replicate correlation, with the R^2^ closer to 1 indicating a stronger correlation between two replicate samples. (C) Enrichment of upregulated genes to KEGG signaling pathway after H. pylori infection, and these genes were involved in signaling pathways such as HIF-1, FoxO, autophagy, AMPK, p53, and mTOR. (D) Enrichment of downregulated genes to KEGG signaling after O-Glycan treatment, and signaling pathways such as p53, oxidative phosphorylation, and cell cycle were significantly downregulated.

To evaluate the effect of O-Glycan on gene expression in gastric epithelial cells induced by persistent H. pylori infection, RNA sequencing (RNAseq) was performed on normal GES-1 cells, H. pylori persistently infected GES-1 cells, and H. pylori-persistently infected GES-1 cells with O-Glycan treatment, respectively. All experiments were performed at least three times. Differentially expressed genes were identified using Fold Change ≥ 1.5 and False Discovery Rate (FDR) < 0.05 as screening criteria. In H. pylori-persistently infected GES-1 cells with O-Glycan treatment, 378 genes were identified with significant changes. There were 235 upregulated genes and 142 downregulated genes in O-glycan-treated GES-1 cells compared to those untreated with O-Glycan (Fig. 6A and B and Table S1). The enrichment analysis showed that persistent H. pylori infection mainly led to the upregulation of various signaling pathways, such as HIF-1, FoxO, autophagy, AMPK, p53, and mTOR (Fig. 6C and Fig. S5A). In the O-Glycan treatment group, the enrichment signals of signaling pathways, including p53, oxidative phosphorylation, and cell cycle, were significantly downregulated (Fig. 6D and Fig. S5B). The results from this study are in agreement with those previously reported that the activation of p53 signaling pathways was observed in both *Mongolian gerbils* and patients infected with H. pylori (21, 22). The results above indicated that the significant downregulation of the p53 signaling pathway after treatment with O-Glycan might be associated with the protective effect of O-Glycan on the H. pylori–induced injury of gastric epithelial cells.

## DISCUSSION

H. pylori infrequently colonizes deep mucosal surfaces in the stomach, possibly because α1,4-GlcNAc-capped O-glycan-rich mucin secreted by glandular cells in the deep mucosa limits H. pylori colonization in the deep mucosa (23, 24). It calls for further verification of whether O-Glycan can reduce the colonization of H. pylori in gastric epithelial cells and inhibit the H. pylori-induced injury of gastric epithelial cells. This study found that O-Glycan significantly suppressed the urease activity and reduced H. pylori adhesion and injury to gastric epithelial cells in the *in vitro* model of persistent H. pylori infection in GES-1 cells. These results not only suggest that O-Glycan may play an important role in the treatment of H. pylori infection, but also provide evidence for its potential clinical application.

As recent studies have demonstrated, H. pylori adhesion to cells is enhanced by releasing CGT through outer membrane vesicles and acting on host cell membrane cholesterol (11, 25). Hsu et al. reported that H. pylori adhesion to cells was attenuated after cells were treated with water-soluble cholesterol (11). Nevertheless, it is easy to trigger cardiovascular diseases with a high cholesterol level, which is unfavorable for clinical application (14, 26). A previous study demonstrated that O-Glycan competitively inhibited the activity of CGT (15). Additionally, this study further proved that O-Glycan suppressed the H. pylori adhesion to gastric epithelial cells. This mucin O-Glycan, secreted by gastric gland cells, holds natural antibacterial activity and is nontoxic and harmless to organisms.

**FIG 7:**
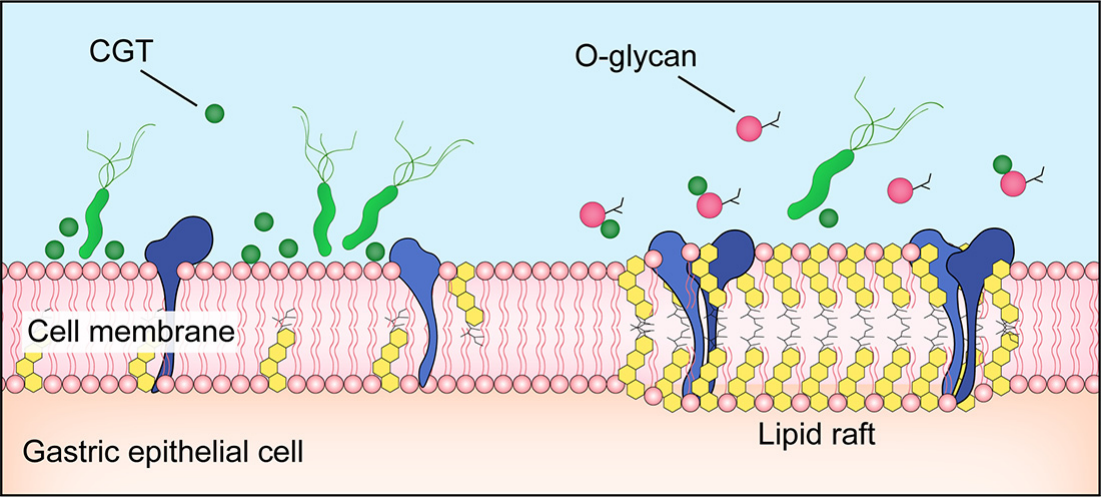
O-Glycan protection of disrupted lipid rafts of gastric epithelial cells infected with H. pylori.

As a nanoscale domain on the cell membrane, lipid rafts are rich in cholesterol and sphingomyelin, contain a variety of receptors, channel proteins, and signaling molecules, and serve as a functional platform for the interactions of protein-protein, protein-lipid, and lipid-lipid (27). Lipid rafts are involved in the regulation of several processes such as pathogen adhesion, cellular signaling, and immune response, and are the key cellular domain mediating H. pylori infection of gastric epithelial cells and causing inflammatory responses (12). H. pylori infection stimulates the recruitment and activation of immune cells such as Th1, in which Th1-dominant CD4^+^ T cells facilitate the inflammatory responses in gastric tissue by secreting IFN-γ (28), thereby playing a decisive role in controlling bacterial load and infection of H. pylori (29, 30). The IFN-γ signaling depends on the integrity of the interferon gamma receptor (IFNGR), whereas the proper assembly of its subunits (IFNGR1 and IFNGR2) relies on intact lipid rafts (31). Referring to the findings by Morey et al. (14), CGT secreted by H. pylori depletes cholesterol, which results in the disruption of lipid rafts on the gastric epithelial cell membrane, thereby impeding the IFN-γ signaling in gastric epithelial cells. This facilitates H. pylori to evade the inflammatory response, which is closely related to H. pylori immune escape and persistent colonization (10, 14). Hence, CGT is considered a potential therapeutic target for the eradication of H. pylori infection (32). Previous studies have proven that O-Glycan competitively inhibits the activity of CGT, while this study observed that O-Glycan diminished the H. pylori-induced disruption of lipid rafts of gastric epithelial cells (Fig. 7). Further investigations are still necessary to clarify whether the restoration of IFN-γ signaling in gastric epithelial cells after O-glycan treatment can facilitate the stomach inflammatory response to get rid of persistent H. pylori infection.

Although most populations infected with H. pylori are asymptomatic, essentially all cases progress to chronic gastritis (11). Among infected individuals, approximately 10% progress to peptic ulcer disease, and 1–3% progress to gastric cancer (33). H. pylori mediates the entry of various virulence factors, including cytotoxin-associated gene A (CagA) and vacuolating toxin A (VacA), into host cells through the type IV secretion system (T4SS) to further regulate the proliferation, apoptosis, autophagy, and other processes of gastric epithelial cells and promote cell injury and carcinogenesis (34, 35). Autophagy, a common biological phenomenon in all eukaryotic cells, is an important process in sustaining cellular homeostasis (36). For both normal and tumor cells, autophagy not only maintains cell survival but also promotes cell death, serving a dual role in tumor suppression and promotion (37). Autophagy is regulated by various signaling pathways, including AMPK/mTOR, I3K/Akt/mTOR, and p53 pathways (38). Several studies have revealed that H. pylori infection induces autophagy in gastric epithelial cells (38–40). p53 is an activator of mTOR, and Tsugawa et al. pointed out that VacA might trigger autophagy in gastric epithelial cells through p53 regulation of the mTOR pathway (41). Moreover, AMP-activated protein kinase (AMPK), stimulated by an elevated AMP/ATP ratio, also increased the autophagy level by regulating the mTOR pathway (42). Tsugawa et al. also identified that VacA induced a decrease in ATP level in gastric epithelial cells, speculating that VacA might initiate autophagy via the AMPK pathway. In this study, we discovered that the p53, AMPK, mTOR, and autophagy pathways were all upregulated in GES-1 cells infected with H. pylori, consistent with previous findings. On the contrary, the p53 pathway was significantly downregulated in GES-1 cells treated with O-Glycan, suggesting that O-Glycan might regulate the autophagy level and relieve the injury in gastric epithelial cells via the p53 signaling pathway, a possible mechanism that is worth further research.

Although we identified the inhibitory effects of O-Glycan on H. pylori adhesion and gastric epithelial cell injury with an *in vitro* infection model in GES-1 cells, there are some limitations in this study. For instance, it requires further verification of whether O-Glycan can inhibit H. pylori infection and colonization *in vivo*.

In conclusion, this study has demonstrated that O-Glycan can inhibit H. pylori adhesion and gastric epithelial cell injury *in vitro* and may serve an important role in suppressing H. pylori infection–mediated carcinogenesis.

## MATERIALS AND METHODS

### Cell culture.

GES-1 cells (Procell) were seeded in the DMEM (HyClone) containing 10% fetal bovine serum (Biosharp) and 1% penicillin-streptomycin (HyClone). Cells were cultured in the 5% CO_2_ incubator at 37°C. The medium used for H. pylori infection was free of penicillin-streptomycin.

### H. pylori culture and infection.

A standard strain of H. pylori ATCC 43504 (MINGZHOUBIO) and H. pylori Sydney strain 1 (SS1) provided by Hebei Medical University were inoculated in the Columbia agar plate (Oxoid), and then cultured at 37°C under microaerobic conditions (5% O_2_, 10% CO_2_, and 85% N_2_) for 48 h. Before infection, H. pylori was cultured in the antibiotic-free DMEM overnight. Then, the H. pylori culture was diluted with DMEM to a suspension at 1.0 × 10^7^ CFU/mL to infect GES-1 cells at a multiplicity of infection (MOI) of 100:1 for 24h.

### Urease content test.

Before H. pylori infection, GES-1 cells were treated with 0.50 μg/mL of O-Glycan (Toronto Research Chemicals) or DMEM for 1 h. GES-1 cells were then infected with H. pylori at an MOI of 100:1 for 24 h. The effect of O-Glycan on the urease content of H. pylori in the *in vitro* infection model was determined according to the method described by Adeniyi et al. (17). Briefly, after H. pylori infection for 24 h, the culture supernatant was removed, the cultures were washed twice with 0.02 M phosphate-buffered saline (PBS, pH 7.4), the urease test reagent containing 0.4% phenol red and 2% urea was added to the cultures and incubated at 37°C for 30 min, and the OD values (OD_600_) were measured at 600 nm with the microplate reader (MD).

$$\text{Relative urease content }\left(\%\right)\;=\;\left({\text{OD}}_{\text{6}00}\text{of Sample}/{\text{OD}}_{\text{6}00}\text{of Control}\right)\;\times \text{ 1}00\%$$

### Cell viability assay.

The O-Glycan-treated or untreated GES-1 cells were seeded into 96-well plates and infected with H. pylori at an MOI of 100 for 24 h, respectively. Then, the cells were incubated with 10 μL of cell counting kit-8 (CCK-8) reagent (Solarbio) for 2 h. The absorbance at 450 nm was read using a Spectrophotometer.

### H. pylori adhesion activity test.

After H. pylori infection of O-Glycan treated or untreated GES-1 cells for 24 h as per the procedures described above, the cultures were washed twice with PBS (pH 7.4). The cultures were then digested with 0.25% trypsin solution (Biosharp). The cell suspensions were serially diluted, plated on Columbia Blood Agar, and cultured under microaerobic conditions for 5 days to count viable H. pylori expressed as CFU.

### Immunofluorescence staining and confocal microscopy.

After *in vitro* infection for 24 h, cells were fixed with 4% paraformaldehyde for 1 h, permeabilized with 0.1% Triton X-100 (Biosharp) for 30 min, and blocked with 1% FBS for 1 h. H. pylori, lipid rafts, cytoskeleton, and nucleus were stained with anti–H. pylori antibody (Biosharp), fluorescein isothiocyanate-labeled cholera toxin subunit B (FITC-CTX-B) (Absin), rhodamine-labeled phalloidin (Biosharp), and 4, 6-diamidino-2-phenylindole (DAPI) (Biosharp), respectively, and fluorescence signals were analyzed using a confocal laser scanning microscope (Carl Zeiss).

### RNA sequencing.

Total RNAs from GES-1 cells were extracted using TRIzol reagent (Invitrogen) and RNA samples were then submitted to Biomarker Technologies for transcriptome sequencing and analysis. According to the sequencing instructions and operation manual, the yield of total RNAs was assessed with Nanodrop (Thermo), the cDNA library was prepared with TruSeq RNA Sample Preparation kit (Illumina), and then the library was sequenced with HiSeq Platform (Illumina) for a 150 bp double-end sequencing. All experiments were performed at least three times. All raw data are uploaded to Sequence Read Archive (SRA, accession: PRJNA874328). Differentially expressed mRNAs were selected for analysis of GO and KEGG pathways.

### Statistical analysis.

The results are expressed as the mean ± standard error of the mean (SEM) of three replicate experiments. SPSS Statistics 20.0 (IBM Corp.) was used for data analysis. One-way analyses of variance (ANOVAs) followed by the Student's *t* test and Tukey’s method were performed using GraphPad Prism8, with statistical significance set at *P* value < 0.05.
