# Supplementary material for: Inhibitory and Injury-Protection Effects of O-Glycan on Gastric Epithelial Cells Infected with Helicobacter pylori
Source: Infect Immun. 2022 Oct 3;90(10):e00393-22. doi: 10.1128/iai.00393-22 (PMC9584294; doi:10.1128/iai.00393-22)
Supplement: Supplemental file 1 — Fig. S1 to S5 and Table S1. Download iai.00393-22-s0001.pdf, PDF file, 0.8 MB [file iai.00393-22-s0001.pdf]

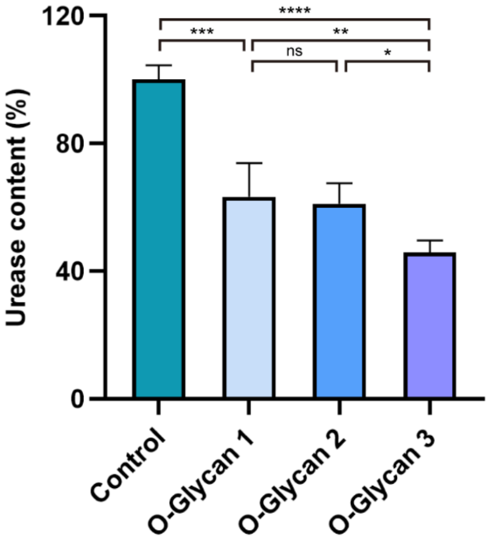

FIG S1. In the *in vitro* infection model, all 3 types of O-glycans significantly reduced the relative urease content of *H. pylori* (SS1) at 50.00 µg/mL, and respectively, the relative urease content was reduced to 63.38%, 61.12%, and 45.90% after the treatment with O-Glycan 1, 2, and 3. The experiments were repeated 5 times for all groups, statistical significance was calculated using a one-way ANOVA test, followed by the Tukey's methods. *, $P < 0.05$, **, $P < 0.01$, ***, $P < 0.001$, ****, $P < 0.0001$, NS, not significant.

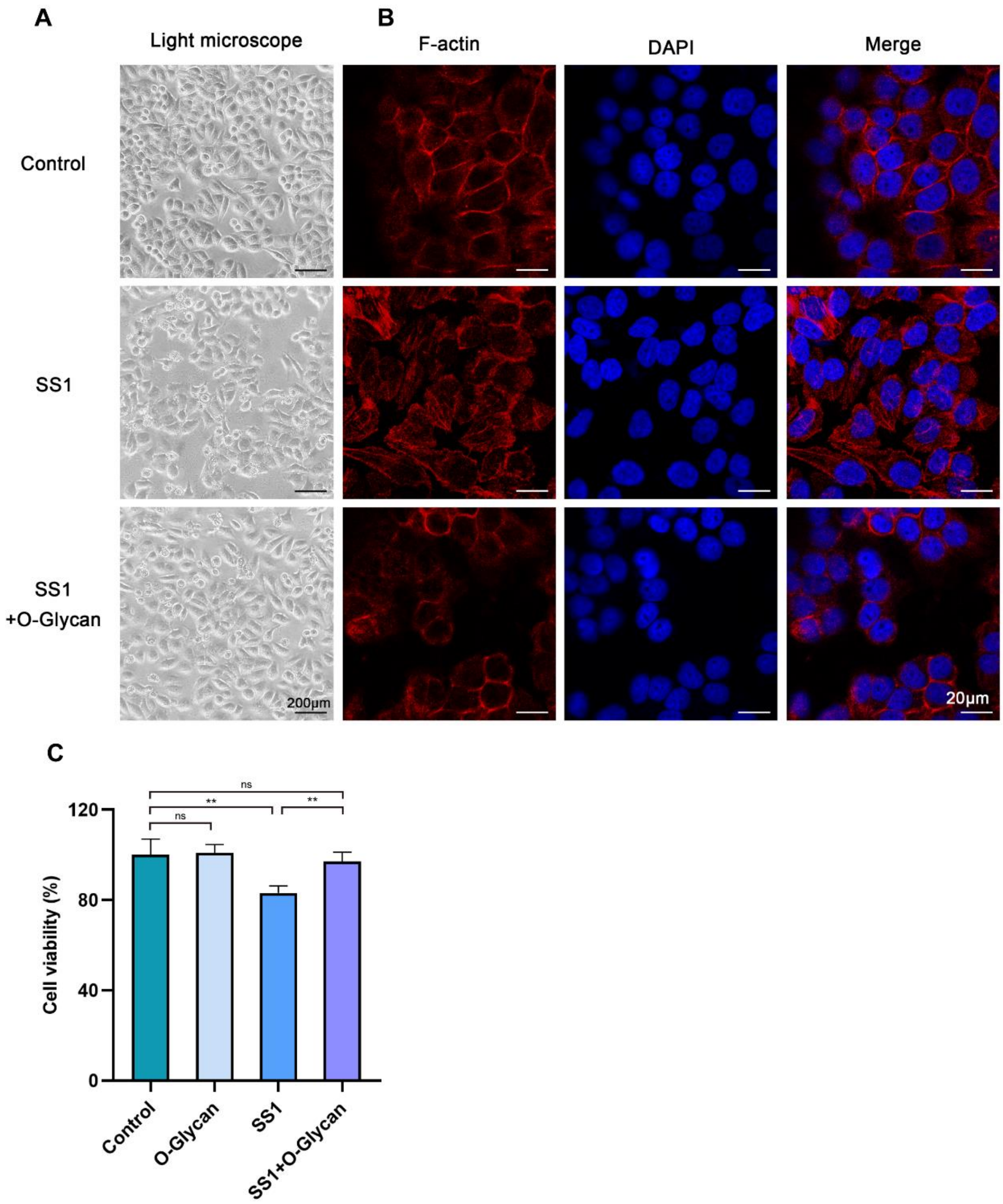

FIG S2. O-Glycan inhibited the injuries of GES-1 cells due to persistent *H. pylori* (SS1) infection. A. Morphological observations at 24 h under the light microscope for normal GES-1 cells, SS1-infected GES-1 cells, and SS1-infected GES-1 cells treated with O-Glycan. Scale bar: 200 μm. B. The cytoskeleton was labeled with F-actin (Red) and the nuclear chromatin was labeled with DAPI (Blue). Fluorescence staining of GES-1 cells in each group was analyzed under the confocal microscope. Scale bar: 20 μm. C. Cell viability of GES-1 after with infected SS1 and treatment of

O-Glycan or not was detected by CCK-8 assay. **, $P < 0.05$, NS, not significant.

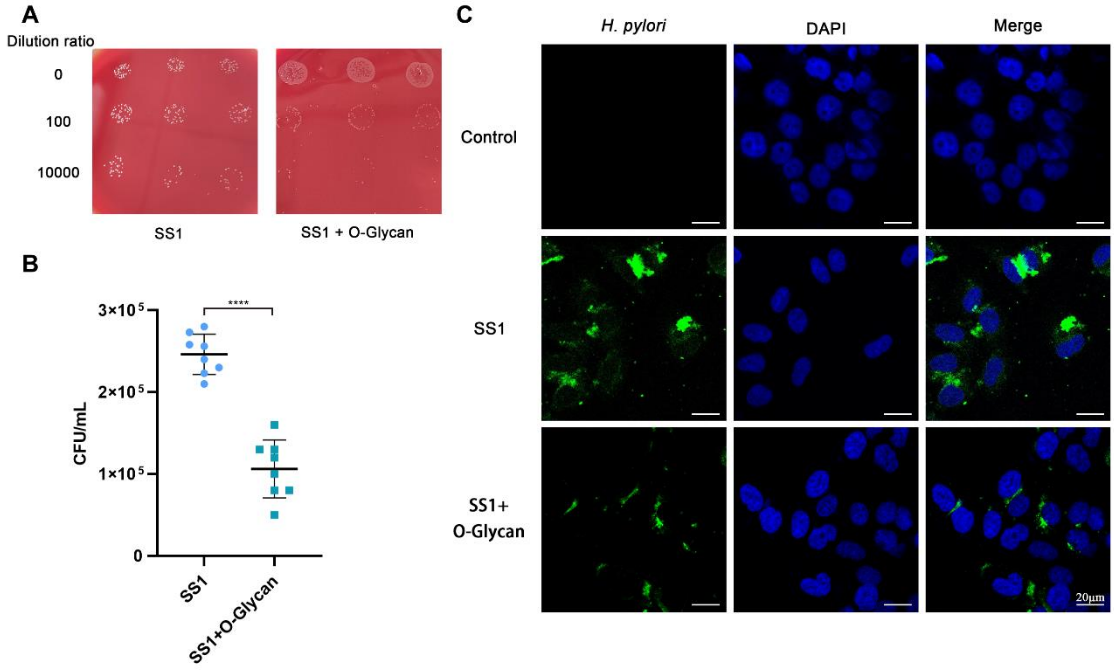

FIG S3. O-Glycan inhibition of *H. pylori* (SS1) adhesion to GES-1 cells. GES-1 cells were untreated or treated with 0.50 μg/mL O-Glycan and then infected with SS1 at an MOI of 100 for 24 h. A. The infected cell lysate was serially diluted onto blood agar plates and incubated for 5 days. Viable *H. pylori* were counted and indicated as colony-forming units (CFUs). B. Colony forming units (CFU)/mL were quantified to evaluate its adhesion to GES-1 cells. The Student's t-test was used to analyze the statistically significant difference between groups. ****$P < 0.0001$. C. SS1 were labeled with anti-*H. pylori* antibody (Green) and nuclei were labeled with DAPI (Blue). Fluorescence signals were analyzed using the confocal microscope. Scale bar: 20 μm.

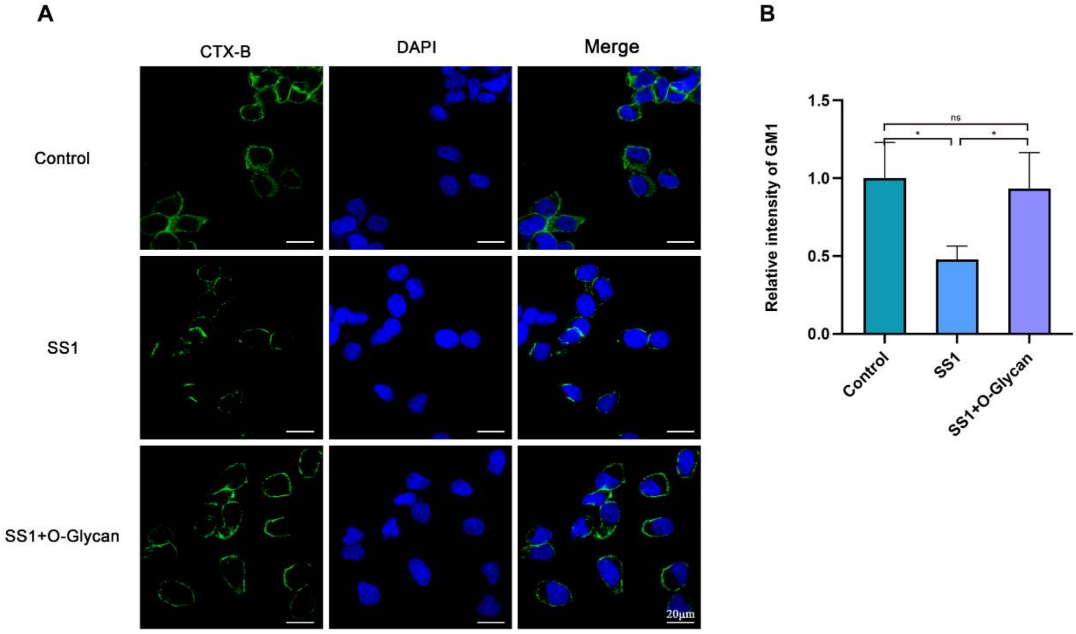

FIG S4. Protective effect of O-Glycan on lipid rafts of GES-1 cells infected with *H. pylori* (SS1). GES-1 cells were untreated or treated with 0.50 μg/mL O-Glycan and then infected with SS1 at an MOI of 100 for 24 h. A. Confocal microscopic analysis of the lipid raft marker GM1 (stained with CTX-B, green) in cellular membranes of cells and DAPI to visualize cell nuclei (blue) . Scale bar: 20 μm. B. Quantitative measurements of relative superficial GM1 signal. For image analyses, was selected from 3 experiments and analyzed by ImageJ software. *, $P < 0.05$; NS, not significant.

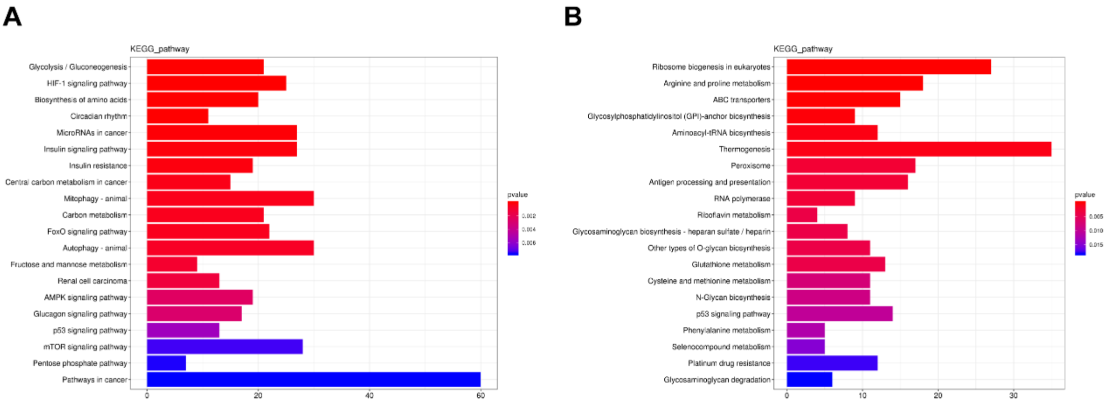

FIG S5. O-Glycan inhibition of upregulation of *H. pylori* infection-mediated inflammation-cancer transformation pathway in GES-1 cells. A. Enrichment of upregulated genes to KEGG

signaling pathway after *H. pylori* infection. B. Enrichment of downregulated genes to KEGG signaling after O-Glycan treatment.

Table S1. Differential gene expression list in *H pylori*-infected GES-1 cells treated or untreated with O-Glycan

Symbol	FDR	log2FC	regulated
EGR1	7.07E-61	1.650181	up
Homo_sapiens_newGene_1339	2.04E-18	1.301549	up
NDUFA4L2	1.10E-68	1.27833	up
SPAG4	1.21E-46	1.233247	up
PPFIA4	5.91E-129	1.215379	up
FAM162A	1.42E-103	1.167781	up
NGLY1	3.72E-29	1.144921	up
RNASE4	2.32E-11	1.060766	up
NKX2-1	1.23E-06	1.051765	up
SEMA5B	2.02E-17	1.038922	up
GTF2IRD2B	2.88E-11	1.026441	up
ALDOC	1.71E-88	1.019317	up
HSF4	1.15E-25	1.017163	up
Homo_sapiens_newGene_24552	2.11E-06	1.004729	up
CAVIN3	5.76E-25	1.004154	up
COL7A1	6.61E-54	0.946992	up
AC074143.1	2.06E-10	0.941078	up
HNF4A	2.76E-10	0.940471	up
ARHGEF6	1.51E-20	0.932357	up
EGLN3	3.58E-16	0.908957	up
KLHL35	3.49E-13	0.908105	up
CA9	5.56E-25	0.907611	up
MEGF6	2.64E-39	0.903205	up
Homo_sapiens_newGene_1340	2.27E-05	0.902284	up
P4HA1	1.91E-45	0.888526	up
EVA1B	7.29E-06	0.887764	up
TCAF2	3.32E-07	0.887341	up
TMEM45A	6.51E-46	0.886769	up
D2HGDH	5.65E-10	0.885387	up
PGK1	4.18E-111	0.885214	up
TMEM158	3.87E-05	0.884188	up
LOX	4.21E-48	0.875662	up
GZMM	0.000153148	0.874934	up
ENDOG	9.09E-06	0.873654	up

Symbol	FDR	log2FC	regulated
PFKFB4	1.33E-56	0.872044	up
PPP1R3E	2.08E-15	0.868319	up
DUSP23	0.000184742	0.865227	up
SH3D21	2.20E-16	0.864409	up
C1QL1	5.53E-13	0.857544	up
KCNK15	1.73E-11	0.853916	up
SEC14L4	9.12E-13	0.85308	up
EFNA3	3.26E-44	0.852821	up
ASL	9.64E-16	0.852382	up
APLN	7.23E-06	0.851248	up
CHRD	3.85E-13	0.850612	up
P4HA2	1.75E-38	0.84968	up
QSOX1	7.87E-59	0.843784	up
C8orf82	6.36E-05	0.841339	up
EFEMP2	8.32E-05	0.840163	up
Homo_sapiens_newGene_13405	2.45E-10	0.839064	up
BNIP3	1.61E-59	0.838455	up
ANGPTL4	2.46E-07	0.828009	up
IDH2	3.05E-36	0.826892	up
CCDC107	3.64E-05	0.820262	up
Homo_sapiens_newGene_13880	3.66E-06	0.818207	up
SLC29A4	1.13E-15	0.817583	up
Homo_sapiens_newGene_19779	1.04E-08	0.815895	up
WDR54	9.60E-15	0.815805	up
GAL3ST1	7.43E-06	0.813476	up
PCK1	1.86E-16	0.811736	up
SLC27A3	5.11E-15	0.811728	up
FSCN2	2.19E-05	0.806781	up
BIRC7	7.08E-09	0.804619	up
IGFBP3	1.67E-39	0.804567	up
BMPER	0.000598705	0.804005	up
NXPH4	1.91E-15	0.799141	up
PDK1	1.19E-20	0.794529	up
KCTD11	1.01E-31	0.792494	up
UPK3BL1	0.001261932	0.791965	up
NOG	5.45E-06	0.788151	up
Homo_sapiens_newGene_3085	2.01E-05	0.787389	up
B3GNT4	4.45E-05	0.781979	up
PTPRN	0.001444355	0.781029	up
NUDT18	3.30E-05	0.780477	up
UCN2	3.61E-06	0.779603	up

Symbol	FDR	log2FC	regulated
COX20	2.01E-09	0.771743	up
GBE1	1.00E-45	0.770908	up
MXI1	1.05E-35	0.770799	up
METTL26	1.31E-12	0.770215	up
GUK1	4.82E-16	0.769275	up
Homo_sapiens_newGene_18448	1.76E-05	0.768424	up
PLOD2	9.46E-41	0.76788	up
PDK3	2.49E-23	0.763347	up
POLE4	7.27E-06	0.761753	up
TMEM256	5.20E-08	0.760145	up
NOXA1	5.97E-07	0.759745	up
Homo_sapiens_newGene_3641	0.000740139	0.758775	up
PPP2R5B	1.93E-09	0.758117	up
GPR146	1.75E-06	0.754966	up
RIMKLA	0.00117923	0.753115	up
PPP1R3G	9.44E-12	0.752432	up
Homo_sapiens_newGene_4892	0.002445607	0.749973	up
GOLGA8N	0.001678076	0.748799	up
MT1E	2.18E-07	0.743021	up
MPI	2.04E-16	0.738886	up
ENO2	1.38E-39	0.738663	up
HILPDA	3.43E-24	0.738282	up
H6PD	2.27E-07	0.735992	up
NREP	1.05E-17	0.735665	up
CFD	1.53E-11	0.733999	up
WDR90	1.06E-28	0.730722	up
HOOK2	1.43E-10	0.728471	up
CHD1L	8.93E-17	0.722029	up
FAM83F	0.003919443	0.720597	up
HNRNPUL2-BSCL2	8.93E-08	0.718376	up
HMGCS1	3.46E-47	0.717769	up
METAP1D	0.001590722	0.717765	up
NUDT14	4.31E-05	0.716725	up
TLCD1	0.000185123	0.716046	up
Homo_sapiens_newGene_9140	9.02E-05	0.715969	up
NOL3	1.49E-21	0.714502	up
BCKDHA	3.02E-17	0.7116	up
CCDC78	0.000154369	0.708566	up
TMEM37	9.93E-07	0.707496	up
RAB4B	0.003140003	0.706835	up
ZMAT1	0.000491761	0.706493	up

Symbol	FDR	log2FC	regulated
SNTA1	1.09E-10	0.706356	up
INSIG2	1.93E-17	0.705794	up
RNF223	2.87E-05	0.705025	up
C4orf3	2.54E-18	0.704349	up
THPO	0.004234027	0.703106	up
Homo_sapiens_newGene_24703	1.96E-07	0.701543	up
HINT2	0.001392304	0.700413	up
IGFBP1	6.40E-10	0.698545	up
DIPK2A	2.11E-10	0.698441	up
ERO1A	5.38E-28	0.697455	up
FAM173A	0.004563157	0.69621	up
KIAA0895L	1.50E-06	0.694589	up
KDM3A	1.55E-30	0.693005	up
ABCB6	0.003541869	0.691765	up
ZNF784	0.002445607	0.691603	up
CRABP2	6.74E-17	0.690953	up
BIK	0.003347597	0.690523	up
PYGL	8.48E-36	0.687586	up
ARID5A	4.98E-05	0.687441	up
CCL28	1.06E-08	0.686741	up
MPG	7.26E-09	0.686101	up
PPP1R14B	2.00E-08	0.683981	up
RPS29	9.23E-09	0.680091	up
JUNB	9.01E-11	0.67835	up
TFR2	1.14E-12	0.678132	up
RHPN1	0.002047867	0.677652	up
DPCD	9.95E-08	0.677006	up
BNIP3L	8.37E-34	0.676975	up
Homo_sapiens_newGene_2168	0.000978601	0.676235	up
CACNA1H	2.64E-13	0.675321	up
ZNF385A	8.57E-16	0.673503	up
PDGFB	1.32E-06	0.672869	up
ELMO3	2.61E-05	0.672076	up
KCNJ6	6.48E-05	0.669588	up
SSBP2	0.000657677	0.668839	up
HYI	0.001805088	0.667874	up
NDNF	0.003055624	0.664605	up
YPEL3	0.000359063	0.662873	up
LRRC56	0.002495299	0.662427	up
HIST1H2BD	5.49E-06	0.660772	up
MXD3	3.46E-05	0.659201	up

Symbol	FDR	log2FC	regulated
FAM229A	0.009478258	0.658906	up
GYS1	1.06E-31	0.657801	up
CYP2D7	0.008773555	0.656279	up
KBTBD11	0.004611492	0.654468	up
F8A2	0.007037764	0.654067	up
PDXP	1.50E-06	0.654049	up
Homo_sapiens_newGene_2604	0.008644538	0.653986	up
AK4	1.48E-29	0.650436	up
KIFC2	2.86E-06	0.649591	up
Homo_sapiens_newGene_1263	4.29E-05	0.64836	up
NENF	0.000185509	0.648357	up
ARHGAP19	4.25E-07	0.647144	up
GOLGA8B	1.45E-18	0.646507	up
HMGCL	0.000165558	0.645592	up
LPIN3	2.15E-12	0.6432	up
TMEM141	1.34E-09	0.641374	up
AC005154.5	0.009413936	0.63796	up
MMP24OS	0.001759858	0.63511	up
CHKB	0.00015331	0.63432	up
ITGA5	1.72E-33	0.633872	up
CCDC85B	8.14E-05	0.632387	up
HOXC4	0.002019291	0.630334	up
Homo_sapiens_newGene_31279	1.61E-37	0.628614	up
C8orf58	9.68E-08	0.627712	up
TRIB2	0.004394317	0.627582	up
Homo_sapiens_newGene_24519	0.003666231	0.626387	up
ATG16L2	0.000139273	0.623857	up
IER5L	2.26E-07	0.623489	up
TRNAU1AP	1.80E-05	0.619481	up
MAFK	6.00E-22	0.615767	up
ANLN	1.26E-29	0.615492	up
ANKRD37	0.000499956	0.615228	up
PRUNE2	2.87E-08	0.61494	up
FUT11	7.37E-21	0.614893	up
HK2	1.75E-29	0.614848	up
ZC3HAV1L	8.92E-07	0.614671	up
PGGHG	0.000891431	0.613998	up
SLC26A6	9.17E-18	0.611085	up
HIST1H1C	1.04E-07	0.611011	up
GPI	8.13E-47	0.610093	up
ACOT1	1.66E-05	0.609687	up

Symbol	FDR	log2FC	regulated
CDK10	1.90E-12	0.608452	up
CCDC189	4.60E-05	0.60758	up
Homo_sapiens_newGene_12788	0.009703771	0.606906	up
ADCK5	0.003056403	0.605989	up
NOTCH3	1.24E-17	0.605584	up
TNNT1	1.42E-07	0.604607	up
TSEN34	3.30E-10	0.603803	up
Homo_sapiens_newGene_3706	0.000405175	0.60364	up
ALS2CL	0.000178147	0.603567	up
RFX2	0.000184771	0.601909	up
Homo_sapiens_newGene_6693	0.009052529	0.600469	up
Homo_sapiens_newGene_7332	2.14E-05	0.600406	up
TPGS1	0.002978155	0.59894	up
FOXL1	1.25E-19	0.598915	up
NDRG1	7.88E-52	0.596941	up
POU5F1	8.40E-10	0.596514	up
AFAP1L1	9.43E-05	0.596127	up
FOXD1	0.002560994	0.595874	up
MZT2B	1.78E-05	0.595425	up
HDHD5	1.71E-08	0.595378	up
LAGE3	0.000185678	0.594485	up
Homo_sapiens_newGene_29271	0.009608687	0.594079	up
VKORC1	2.84E-12	0.593756	up
CCDC167	0.006234461	0.593427	up
ADSSL1	4.10E-10	0.593302	up
Homo_sapiens_newGene_29270	2.39E-05	0.591397	up
ARFGEF3	2.11E-14	0.590876	up
ALKBH7	1.52E-06	0.5905	up
ACSS2	1.88E-20	0.590018	up
EEF1AKMT3	0.00026689	0.589604	up
FBXL8	0.00149176	0.588998	up
AMDHD1	0.000889733	0.588868	up
TPI1	3.86E-52	0.588656	up
Homo_sapiens_newGene_22754	0.00115889	0.587984	up
ARHGEF37	5.02E-05	0.587907	up
TRPT1	0.001258443	0.58728	up
Homo_sapiens_newGene_5914	0.003735687	0.587075	up
CYP26B1	0.000416506	-0.58559	down
PAQR3	0.000421004	-0.58599	down
MT-CO1	2.70E-51	-0.58599	down
GCNT2	9.79E-09	-0.59192	down

Symbol	FDR	log2FC	regulated
ZIC5	3.66E-05	-0.59309	down
RFX3	2.72E-06	-0.59474	down
MT-CO3	8.53E-25	-0.59538	down
VGLL3	0.003801317	-0.59745	down
MCC	4.43E-07	-0.59886	down
LIMA1	2.71E-13	-0.59979	down
TM4SF1	2.02E-33	-0.59994	down
C6orf106	6.89E-30	-0.60401	down
THBS1	6.85E-08	-0.60501	down
PRKCH	3.98E-06	-0.60547	down
MAPRE2	1.17E-07	-0.60573	down
CDK6	1.01E-43	-0.60609	down
TSHZ1	0.003465545	-0.6064	down
SLC7A11	3.40E-06	-0.60913	down
STK10	0.000313745	-0.61003	down
RPL36A-HNRNPH2	0.001881116	-0.61228	down
RNF182	5.79E-26	-0.61337	down
RRS1	3.95E-06	-0.61352	down
Homo_sapiens_newGene_21342	0.006517732	-0.61729	down
ZNF132	0.008956699	-0.61829	down
MARS2	0.000422668	-0.62042	down
DAGLA	0.002054525	-0.62081	down
MT-ND4L	1.19E-40	-0.62236	down
RBMS1	1.60E-07	-0.6232	down
NUAK2	5.35E-05	-0.62379	down
ZBED1	1.49E-16	-0.62426	down
NT5C2	9.26E-18	-0.62469	down
LCN10	0.009579509	-0.62574	down
GAS7	0.006107851	-0.62759	down
OTUD7A	0.006460258	-0.62764	down
Homo_sapiens_newGene_23587	0.000456836	-0.62788	down
MCTP2	0.006815298	-0.62908	down
VDR	0.000153393	-0.62918	down
FOXN1	0.001000645	-0.62925	down
SLC16A7	0.000511981	-0.62944	down
ZNF460	0.008662621	-0.63072	down
GPRIN3	0.001046438	-0.6309	down
TWNK	1.80E-08	-0.63296	down
FAM129A	1.30E-06	-0.63363	down
GLIS3	5.76E-12	-0.63455	down
ANKRD33B	2.11E-14	-0.63578	down

Symbol	FDR	log2FC	regulated
B4GALT6	0.008501893	-0.63579	down
ADAT1	4.00E-06	-0.63591	down
XAGE2	0.009488748	-0.63737	down
MT-CYB	2.12E-39	-0.6388	down
SAMHD1	4.05E-07	-0.63918	down
Homo_sapiens_newGene_13566	0.002498251	-0.64028	down
JAM3	0.00689373	-0.64091	down
LAMA1	1.13E-08	-0.64115	down
SLC27A2	0.000422874	-0.64342	down
TMEM33	2.35E-14	-0.64358	down
CSF2RA	9.12E-07	-0.64419	down
KRT80	1.68E-21	-0.64529	down
ZDHHC11B	0.002047867	-0.64613	down
Homo_sapiens_newGene_20427	0.000667155	-0.64753	down
EMP1	0.000537294	-0.64818	down
GFOD1	7.63E-05	-0.6484	down
HPSE	0.00623142	-0.65075	down
ENG	4.03E-15	-0.652	down
PAPPA	0.000352712	-0.65204	down
PRICKLE1	0.0054447	-0.6534	down
SLC35F2	4.25E-05	-0.65369	down
RFK	1.90E-12	-0.65445	down
TGFB2	1.74E-05	-0.65549	down
Homo_sapiens_newGene_18753	5.05E-09	-0.65605	down
GPR37	0.00010255	-0.65908	down
OGFOD1	1.72E-07	-0.65989	down
Homo_sapiens_newGene_23323	0.001845695	-0.66231	down
MMP15	2.07E-05	-0.66642	down
EDNRA	0.000626193	-0.66806	down
ZNF585B	0.000853078	-0.6691	down
AFF1	6.71E-36	-0.68142	down
Homo_sapiens_newGene_28320	3.83E-05	-0.68178	down
Homo_sapiens_newGene_451	0.006474311	-0.68374	down
SLC25A12	1.78E-05	-0.68843	down
NCALD	0.000100695	-0.68937	down
RAB3IL1	1.11E-07	-0.68963	down
DNAJC6	6.69E-11	-0.69023	down
PMAIP1	2.83E-06	-0.69136	down
C1orf109	3.06E-09	-0.6963	down
SLC22A15	0.000191574	-0.69795	down
OLFML1	0.001468307	-0.70482	down

Symbol	FDR	log2FC	regulated
ZMPSTE24	1.59E-21	-0.70566	down
KBTBD6	2.53E-08	-0.70842	down
AC138894.1	0.001064823	-0.71216	down
BCL2	0.000291109	-0.71497	down
SRGAP1	7.69E-16	-0.71548	down
NEU3	4.33E-09	-0.71855	down
ADGRG1	3.46E-13	-0.72279	down
CAVIN4	0.002869616	-0.72322	down
TFRC	1.61E-59	-0.72465	down
ABCC2	1.75E-29	-0.72531	down
ENDOD1	0.001935665	-0.72863	down
Homo_sapiens_newGene_31434	1.32E-08	-0.73081	down
TRIML2	0.002069946	-0.73474	down
SLC27A4	6.52E-10	-0.73544	down
TMEM206	0.000158203	-0.74403	down
SYNE1	6.73E-34	-0.75115	down
CYFIP2	0.000123294	-0.75719	down
GADD45A	1.91E-05	-0.75814	down
RAB29	8.87E-14	-0.75814	down
MT-ATP6	1.58E-44	-0.75916	down
GPD1L	2.45E-06	-0.76364	down
GNB1L	1.36E-06	-0.76416	down
CCND1	7.95E-13	-0.76519	down
TNFAIP3	4.58E-25	-0.76932	down
SEMA3C	2.45E-19	-0.77533	down
LIFR	1.75E-47	-0.77612	down
EPB41L3	0.000294839	-0.77888	down
TBC1D30	0.0003582	-0.78614	down
OLR1	2.60E-09	-0.78786	down
MAMLD1	1.30E-07	-0.78849	down
NRXN3	0.001049046	-0.78891	down
MITF	5.89E-19	-0.79165	down
Homo_sapiens_newGene_30336	0.000877847	-0.79331	down
HECW1	3.29E-09	-0.79662	down
TMEM178B	1.73E-09	-0.80848	down
MEGF9	1.29E-39	-0.81073	down
CDKL1	3.93E-05	-0.81535	down
HRK	2.93E-05	-0.81872	down
DOK7	1.94E-05	-0.82621	down
SHISA2	0.000535353	-0.82762	down
SETDB2	1.01E-18	-0.82804	down

Symbol	FDR	log2FC	regulated
SLC7A2	8.75E-35	-0.83023	down
MT-ATP8	5.51E-40	-0.83887	down
STON2	1.48E-06	-0.85639	down
VSIR	2.16E-06	-0.87225	down
MT-CO2	2.30E-81	-0.89993	down
SH3RF3	6.96E-09	-0.91176	down
MOB3B	2.88E-12	-0.92775	down
ABCA13	2.03E-10	-0.92999	down
PSG4	5.83E-08	-0.991	down
SPINK5	2.27E-37	-0.99225	down
PAPPA2	3.83E-52	-1.0484	down
ALPK2	3.40E-22	-1.15165	down
SEMA5A	5.23E-25	-1.16649	down
GCNT3	4.78E-16	-1.1816	down
C15orf38-AP3S2	6.01E-14	-1.4542	down
Homo_sapiens_newGene_13874	2.60E-28	-1.50661	down

44
